# Fluent but not fail-safe: machine-translated radiology reports still need expert review

**DOI:** 10.1186/s41747-026-00827-7

**Published:** 2026-10-14

**Authors:** Constantin Seibold, Timo Leichenich, Patrick Stein, Edem Atsiatorme, Patrick Kliesch, Gökçe A. Sevim, Peter Trauth, Alexander Kremer, Arved Bischoff, Hans-Ulrich Kauczor, Matthias A. Fink

**Affiliations:** Clinic for Diagnostic and Interventional Radiology, University Hospital Heidelberg, Heidelberg, Germany

**Keywords:** Artificial intelligence, Language translation, Medical reports, Patient safety, Radiology

## Abstract

**Objectives:**

Cross-border care, teleradiology, and international referral pathways increasingly require radiology report translation, but isolated mistranslations may alter clinical meaning. We evaluated German-to-English machine translation of radiology reports and correction-based localization of text requiring review.

**Materials and methods:**

In this retrospective study, 310 de-identified German radiology reports from June and July 2025 were translated into English with DeepL. Eight radiologists assigned 5-point quality scores and corrected perceived errors. Thirty reports were reviewed by all eight readers, and 280 reports were assigned for single-reader evaluation, yielding 520 evaluations. Automated metrics and large language model judges were tested for report-level identification of translations requiring review; a correction-based segment-level edit-prediction model was evaluated on held-out shared reports.

**Results:**

Most translations were rated good or excellent (339/520, 65.2%), but clinically important mistranslations involved diagnosis, anatomy, and radiologic terminology. Agreement was low for ordinal quality scores (Krippendorff α = 0.078, *p* = 0.006) but high for expert corrections (pairwise “bilingual evaluation understudy”–BLEU = 83.0, *p* < 0.001). In the conservative primary screen of 30 shared reports, no automated method discriminated translations requiring review (area under receiver operating characteristic curve [AUC] = 0.51–0.65; *p* = 0.100–0.920). The final edit-prediction model achieved a concordance index of 0.697 and an AUC of 0.731 for segments edited by at least six of eight readers.

**Conclusion:**

Machine-translated radiology reports were often acceptable on average, but translations containing errors requiring expert review persisted. Expert corrections were more reproducible than ordinal quality scores and supported localization of text requiring human review.

**Key Points:**

***Question*** Cross-border radiology workflows increasingly require report translation, but fluent machine translations may still contain meaning-changing errors requiring expert review for safe care.

***Findings*** Most machine translations were rated good or excellent, yet clinically important mistranslations occurred, and automated report-level metrics did not reliably identify reports needing review.

***Relevance statement*** Machine translation may support cross-border radiology workflows, but clinically important mistranslations in otherwise fluent reports require human-in-the-loop oversight and tools that localize text requiring expert review.

**Graphical Abstract:**

**Figure:**
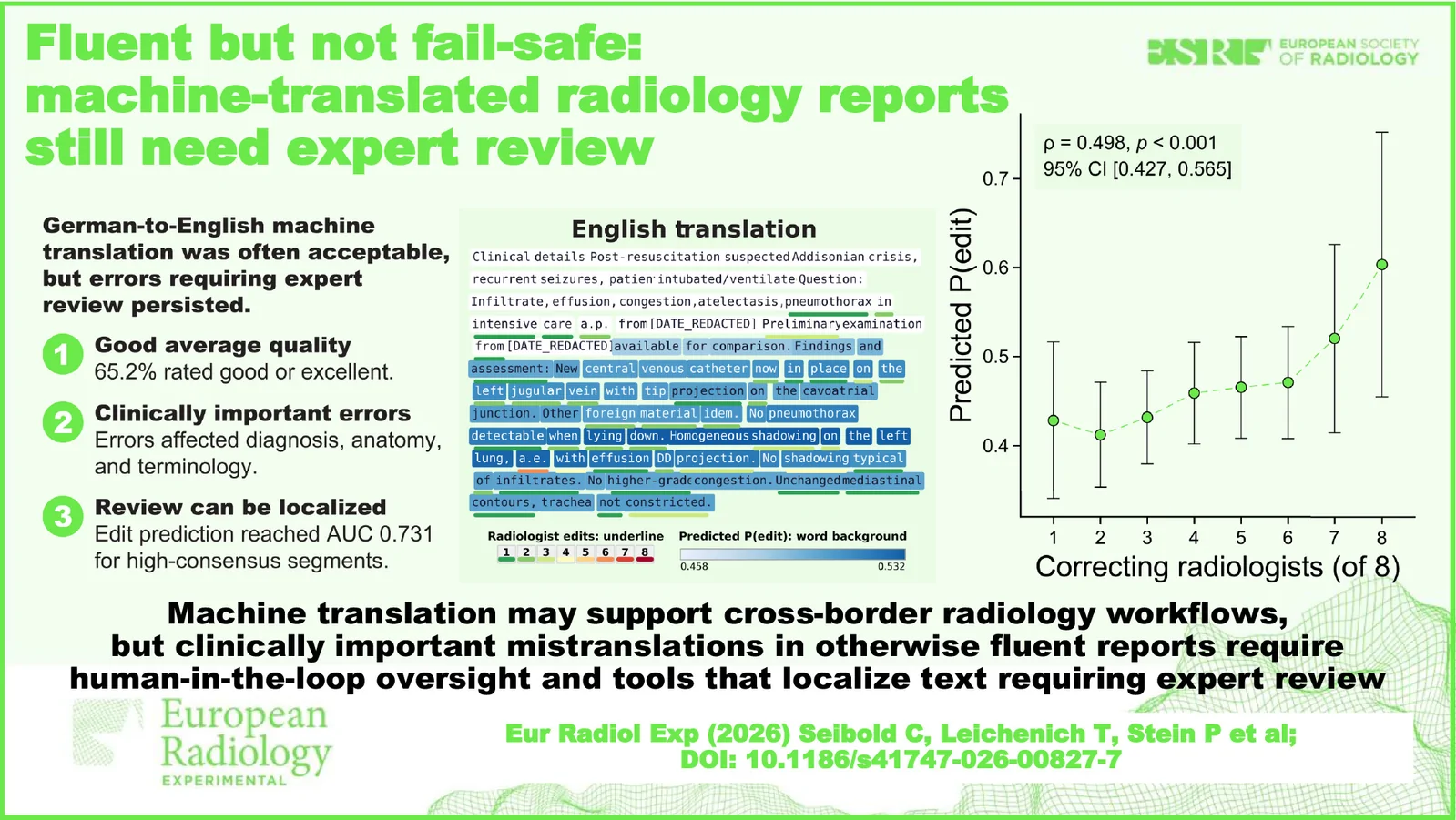

## Background

Radiology reports increasingly cross language boundaries because of teleradiology, international referral pathways, cross-border care, and outsourcing of reporting services [1, 2]. In these settings, translation of radiologic findings, impressions, and recommendations may be needed for clinical decision-making, follow-up planning, and communication between institutions. Machine translation (MT) and large language models (LLMs) have practical appeal for transforming radiology reports into alternative textual outputs [3–5]. However, radiology reports are dense clinical documents. They contain domain-specific terminology, abbreviations, laterality markers, negation, uncertainty, and management-relevant phrasing, such that even a single mistranslation may alter the interpreted diagnosis, anatomy, or urgency of follow-up [6, 7].

This safety concern extends beyond translation to the broader use of LLMs for radiology text processing. Recent work has evaluated LLM-generated simplified radiology reports for patient communication [8, 9], extraction of structured information from free-text stroke computed tomography (CT) reports [10], and interpretation of brain CT referrals according to clinical imaging guidelines [11]. These studies show that LLM-based text transformation may support radiology workflows, but they also underscore the need for task-specific instructions, expert evaluation, and clinically meaningful validation rather than reliance on fluent output alone. More broadly, differences between technical and clinical perspectives on AI validation highlight the risk that aggregate performance measures may not fully address safety-critical clinical use cases [12].

Automated quality assessment remains challenging in this setting. Conventional MT metrics such as BLEU, chrF + +, BERTScore, and the cross-lingual optimized metric for evaluation of translation (COMET) can correlate with human judgment at the system level, and reference-free quality-estimation methods have expanded the available toolkit [13–19]. However, these methods primarily capture overall similarity or average quality and may miss rare but clinically consequential errors within otherwise fluent reports. Human evaluation is also not straightforward: ordinal quality scores are easy to collect but may reflect reader-specific calibration and tolerance for stylistic variation, whereas post-edit corrections may more directly identify the wording that experts consider clinically or linguistically necessary to change [20].

The unmet need is therefore not only to determine whether machine-translated radiology reports are good on average, but to determine whether clinically relevant errors can be identified and localized in a way that supports safe workflow integration. The purpose of this study was to evaluate the clinical safety of German-to-English machine-translated radiology reports using expert radiologist review. We assessed radiologist quality scores and post-edit corrections, evaluated automated report-level methods for identifying translations requiring review, and tested whether correction-based segment-level modeling could localize text requiring expert verification.

## Methods

**Fig. 1:**
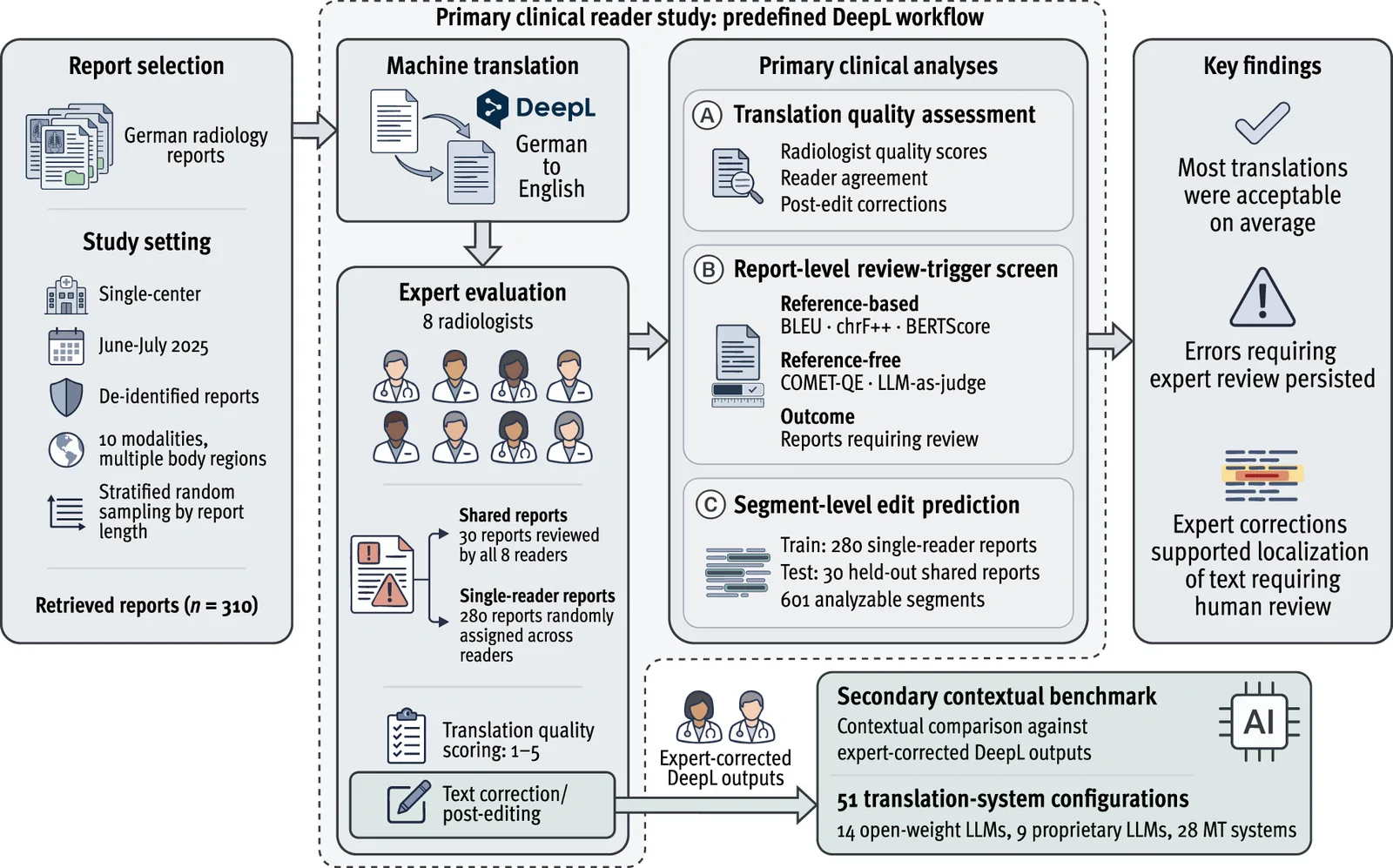
Study design and analytical workflow. The flow chart summarizes the primary clinical reader study and the secondary contextual benchmark. A total of 310 de-identified German radiology reports were selected using stratified random sampling by report length and translated from German to English with DeepL. This predefined DeepL workflow constituted the primary clinically evaluated translation pipeline. Eight radiologists independently assessed translation quality on a 5-point scale and performed text correction/post-editing. Thirty shared reports were reviewed by all eight readers, whereas 280 additional reports were randomly assigned for single-reader evaluation. Primary clinical analyses included radiologist quality assessment, report-level review-trigger screening using reference-based and reference-free automated methods, and segment-level edit prediction. Separately, expert-corrected DeepL outputs were used as reference texts for a secondary contextual benchmark of 51 translation-system configurations, including 14 open-weight LLM configurations, nine proprietary LLM configurations, and 28 dedicated machine-translation (MT) configurations. This benchmark was intended to contextualize the clinically evaluated DeepL workflow and was not interpreted as a direct clinical safety ranking. Key findings are summarized on the right

This retrospective study used de-identified radiology reports and was approved by the institutional review board of University Hospital Heidelberg. Informed consent was waived because the corpus was retrospective and de-identified. The report was the unit of sampling and expert evaluation. The overall study flow is summarized in Fig. 1.

### Report sample and MT

German radiology reports from June and July 2025 were selected from a de-identified institutional text corpus by stratified random sampling across report-length strata. The final sample comprised 310 reports spanning ten imaging modalities and multiple body regions (Table 1). The reports reflected routine institutional practice and included 37 structured and 273 primarily free-text reports. Standard section headings, template elements, and report-internal abbreviations were retained when present. Report length ranged from 34 to 1,529 words (median, 136 words), and patient-level demographic data were not extracted. Dates, patient names, and other directly identifying information were removed before translation; no abbreviation expansion, terminology normalization, or other manual preprocessing was performed.

**Table 1:** Study sample and evaluation characteristics

Parameter	Value
Unique radiology reports, *n*	310
Total expert evaluations, *n*	520
Shared reports evaluated by all 8 readers, *n*	30
Primary reports evaluated by 1 reader, *n*	280
Analyzable 16-token segments in shared test set, *n*	601
Report length, median (range), words	136 (34–1529)
Imaging modalities represented, *n*	10
Quality score 4–5 (good or excellent), *n*/*N* (%)	339/520 (65.2)
Quality score 1–2 (poor or unusable), *n*/*N* (%)	58/520 (11.2)

Study characteristics are reported per unique report unless otherwise indicated. Quality scores are reported per expert evaluation and were assigned on a 5-point scale: 1 = unusable, 2 = poor, 3 =  adequate, 4 = good, and 5 = excellent

DeepL was selected a priori because it is widely used in European professional settings, supports high-resource German-to-English translation, and represents a readily available system relevant to cross-border radiology workflows. All reports were translated as complete documents using the DeepL Application Programming Interface (API) v2 Free tier (DeepL SE, Cologne, Germany) on January 27, 2026, with the source language set to German and the target language to English. The translated text comprised all available report components, including clinical indication, technique/acquisition information, findings, and impression/conclusion sections when present. No custom glossary was used, and default API settings were retained for formality, sentence splitting, formatting preservation, model type, and tag handling. Because DeepL does not expose a fixed public model version for each request, the API access modality and translation date are reported as reproducibility anchors. Only de-identified reports were transmitted, and no additional data-protection configuration was used. This predefined DeepL workflow formed the basis of the clinical reader study; additional MT and LLM systems were assessed separately in a contextual benchmark against expert-corrected DeepL outputs.

### Radiologists’ evaluation and reference corrections

Eight radiologists independently compared each German source report with its English DeepL translation. The group comprised two attending radiologists and six radiology residents, with a mean radiology experience of 3 years (range 0.8–8.0 years). German was the clinical working language of all readers. English proficiency was defined as self-reported professional working proficiency based on regular use of English for scientific literature and clinical or scientific communication; no standardized language test was administered.

For each report, readers assigned an overall translation quality score on a 5-point scale (1, unusable; 2, poor; 3, adequate; 4, good; and 5, excellent) and corrected wording they considered inaccurate or in need of revision. Each radiologist evaluated the same 30 shared reports plus 35 reports assigned from the remaining 280, yielding 65 reports per reader and 520 evaluations overall. Reports were presented in randomized order, and evaluations were completed independently and self-paced through the study annotation interface, with pauses and multiple sessions permitted. Readers knew that the task concerned translation quality but were blinded to other readers’ assessments and to all automated metrics, LLM-judge, benchmark, and model outputs. Both the German source and English translation were displayed because direct comparison was required. Previous evaluations could be revised before final submission. Limiting the workload to 65 reports per reader and permitting pauses were used to reduce fatigue.

### Contextual benchmark of translation systems

To contextualize the clinically evaluated DeepL workflow, we benchmarked 51 translation-system configurations using expert-corrected DeepL translations as reference texts. The benchmark comprised nine proprietary LLM configurations, 14 open-weight LLM configurations, and 28 dedicated machine-translation (MT) configurations. Reference-based performance was assessed using BLEU, chrF++, BERTScore, and COMET, and reference-free quality estimation using COMET quality estimation (COMET-QE). A reference-free edit-prediction score was additionally included for descriptive contextual benchmarking. Because the reference texts were post-edits of DeepL translations rather than independently produced de novo translations, the benchmark was interpreted as a comparison of similarity to expert-corrected DeepL-style translations, not as a definitive head-to-head ranking of clinical translation safety. The evaluated systems and their prompting and inference settings are detailed in Supplementary Tables S1 and S2. Complete LLM prompts are provided in Appendix A, and the multidimensional quality metrics-style error-annotation guideline in Appendix B.

### Report-level screen for translations requiring review

Report-level analyses were performed in the 30 shared reports to determine whether automated methods could identify translations requiring expert review. The conservative primary review-trigger endpoint was positive when at least one reader assigned an overall quality score of 2 or lower, because a single poor or unusable rating may be sufficient to prompt manual review in a safety-oriented workflow. A sensitivity analysis applied a stricter threshold requiring ratings of 2 or lower from at least two readers.

The evaluated methods included BLEU, chrF++, BERTScore, COMET-QE, two reference-free LLM judges (Claude Sonnet 4 and GPT-5.1), and mean edit rate as a reader-derived comparator. For the LLM-as-judge analyses, each model received the German source report and corresponding DeepL translation without an expert-corrected reference. The extensible markup language output was parsed automatically, and the numeric <quality> value was used as the report-level judge score. For receiver operating characteristic analyses, judge scores were reoriented so that higher values indicated a greater likelihood of requiring expert review.

### Correction-based segment-level edit prediction

To localize text likely to require human revision, German source reports and English DeepL translations were partitioned into overlapping 16-token windows with 50% overlap. Sixteen-token windows were selected as the primary analysis unit because they retained significant multi-reader edit signal while allowing phrase-level localization (Supplementary Table S3). Thirty-two-token windows were additionally generated for cross-scale averaging to reduce boundary effects and improve robustness. Corresponding German and English segments were embedded using multilingual E5 and concatenated into a 4096-dimensional feature vector.

A multilayer perceptron was trained to predict whether a translated segment would be edited by a radiologist. Training comprised supervised contrastive pretraining with clinically informed synthetic perturbations, followed by fine-tuning on expert correction labels. Synthetic perturbations represented plausible translation failures, including terminology substitutions, abbreviation errors, anatomic or laterality changes, altered negation or uncertainty, numerical changes, and unrelated or untranslated text. Ill-fit negative pairs, generated by pairing German source segments with unrelated English or German text, were included to improve robustness to degenerate translation output.

**Fig. 2:**
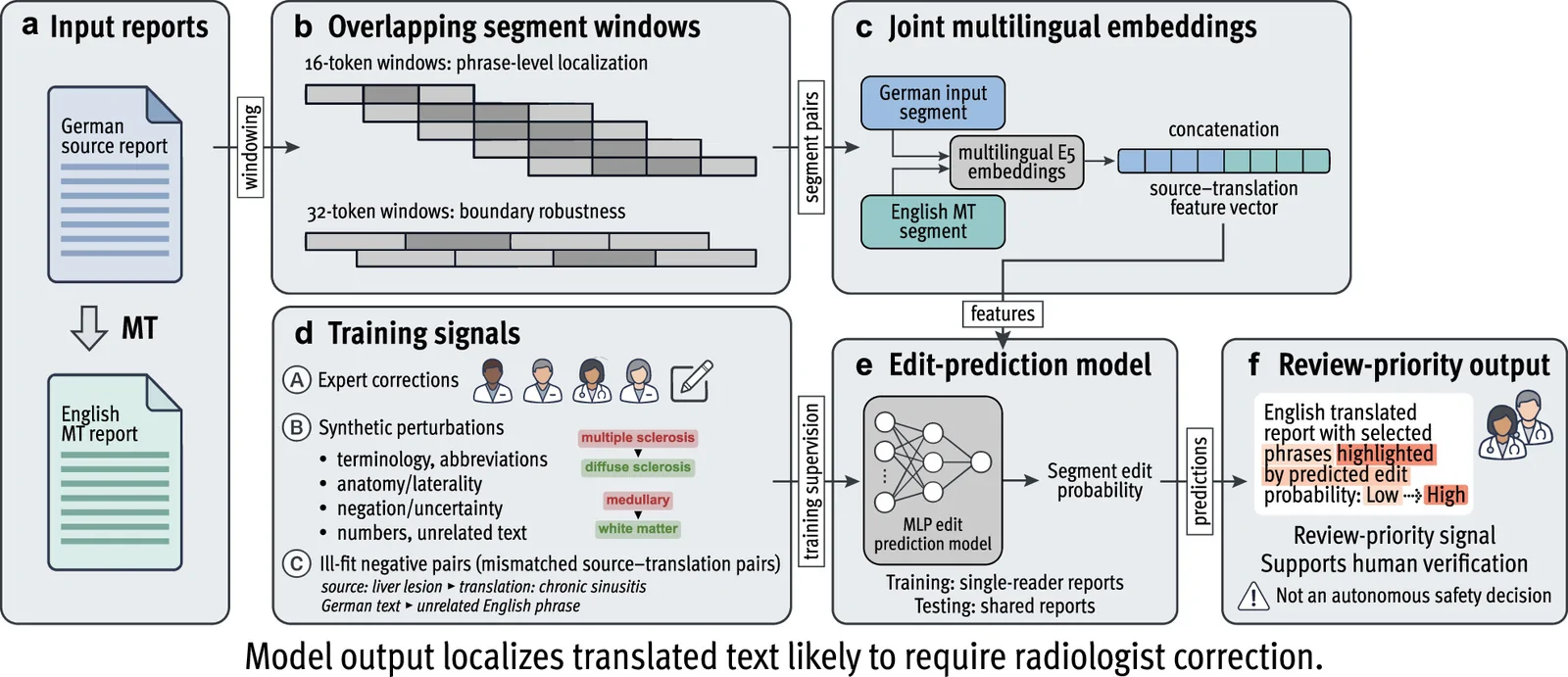
Segment-level edit-prediction workflow for localizing translated text requiring review. **a** German source reports and their English machine translations served as paired inputs. **b** Both texts were partitioned into overlapping 16-token windows for phrase-level localization and 32-token windows for cross-scale averaging. **c** Paired German and English segments were embedded with multilingual E5 and concatenated into a joint source-translation feature vector. **d** Training used expert corrections, clinically informed synthetic perturbations, and ill-fit negative pairs as supervision. **e** A multilayer perceptron generated segment-level edit probabilities. **f** These probabilities were interpreted as review-priority signals to support human verification rather than as autonomous safety decisions

Model development used the 280 single-reader reports with fivefold cross-validation. Held-out testing was performed exclusively on the 30 shared reports, comprising 601 analyzable 16-token segments. For each segment, the number of radiologists who edited it, ranging from 0 to 8, served as the multi-reader consensus target. Model output was interpreted as a review-priority signal for human verification rather than as an autonomous safety decision. The segment-level workflow is summarized in Fig. 2.

### Statistical analysis

Inter-reader agreement for ordinal quality scores was assessed in the shared reports using Krippendorff α and a two-way random-effects, absolute-agreement, single-measure intraclass correlation coefficient (2,1). Agreement between expert-corrected translations was summarized as the mean of per-report pairwise BLEU scores between reader-corrected versions; the inter-reader human ceiling used in the contextual benchmark was calculated using corpus-level BLEU aggregation. Benchmark stability across reader references was assessed by variance decomposition and Kendall’s coefficient of concordance. Report-level discrimination was quantified using the area under the receiver operating characteristic curve (AUC), with bootstrap 95% confidence intervals (CIs) based on 2,000 resamples and *p*-values obtained by permutation testing with 10,000 permutations. Segment-level edit-prediction performance was assessed using the ordinal concordance index and AUCs for segments edited by at least four and at least six of eight readers. Because the inferential target was segment-level localization, bootstrap CIs were calculated by resampling windows. Incremental model variants were compared with the baseline using paired bootstrap tests. Associations between automated measures, reader assessments, and edit consensus were assessed using signed Spearman rank correlations. For report-level correlations, 95% CIs and two-sided *p*-values were derived from the same 2,000-resample bootstrap distribution. Segment probabilities were aggregated to the report level as the proportion of windows exceeding the Youden threshold. All tests were two-sided, with *p* < 0.05 considered statistically significant.

## Results

### Report sample and expert evaluation

**Fig. 3:**
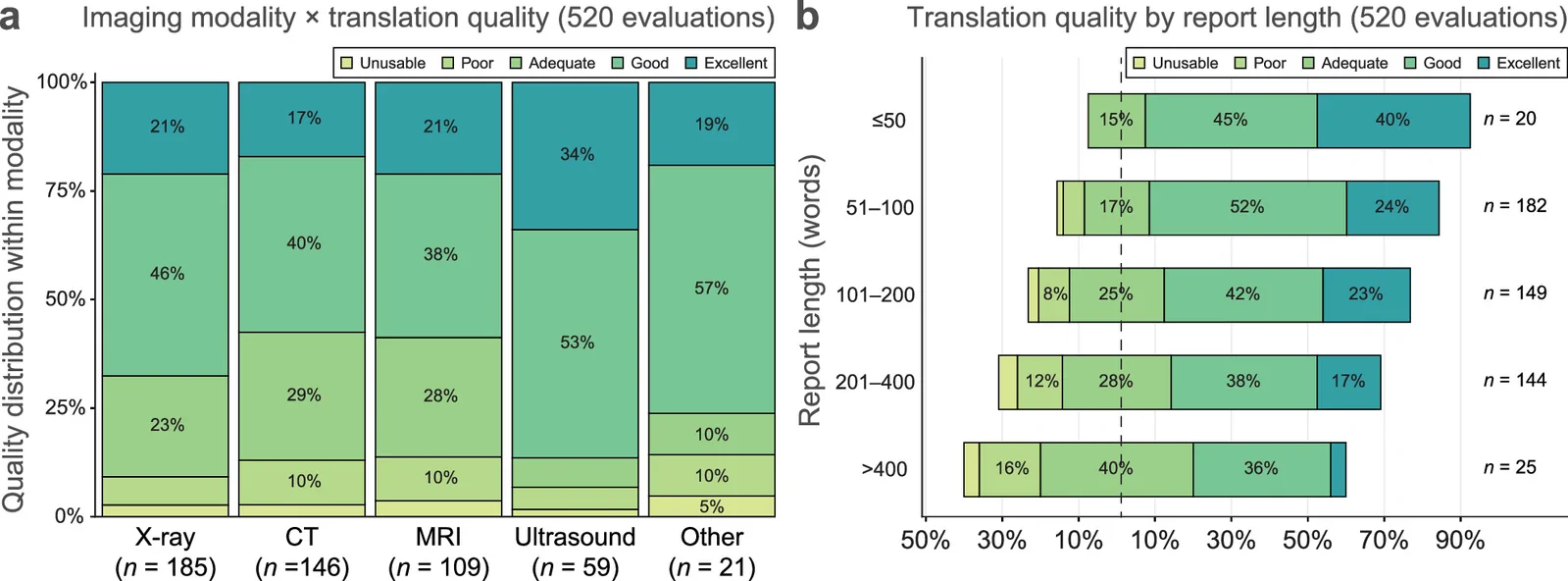
Translation quality stratified by imaging modality and report length. **a** Distribution of expert translation quality scores within imaging modality categories across 520 evaluations. **b** Distribution of expert translation quality scores according to report length across 520 evaluations. Report length categories are ≤ 50, 51–100, 101–200, 201–400, and > 400 words. Quality scores were assigned on a 5-point scale: 1 = unusable, 2 = poor, 3 = adequate, 4 = good, and 5 = excellent

The study included 310 de-identified German radiology reports spanning ten imaging modalities and multiple body regions (Figs. 1 and 3, and Table 1). All reports were translated from German to English with DeepL. Thirty reports were evaluated by all eight radiologists, and 280 additional reports were randomly assigned for single-reader evaluation, yielding 520 total expert evaluations. The shared reports remained for multi-reader analyses and held-out segment-level testing, corresponding to 601 analyzable 16-token segments.

### Translation quality and clinically important mistranslations

**Fig. 4:**
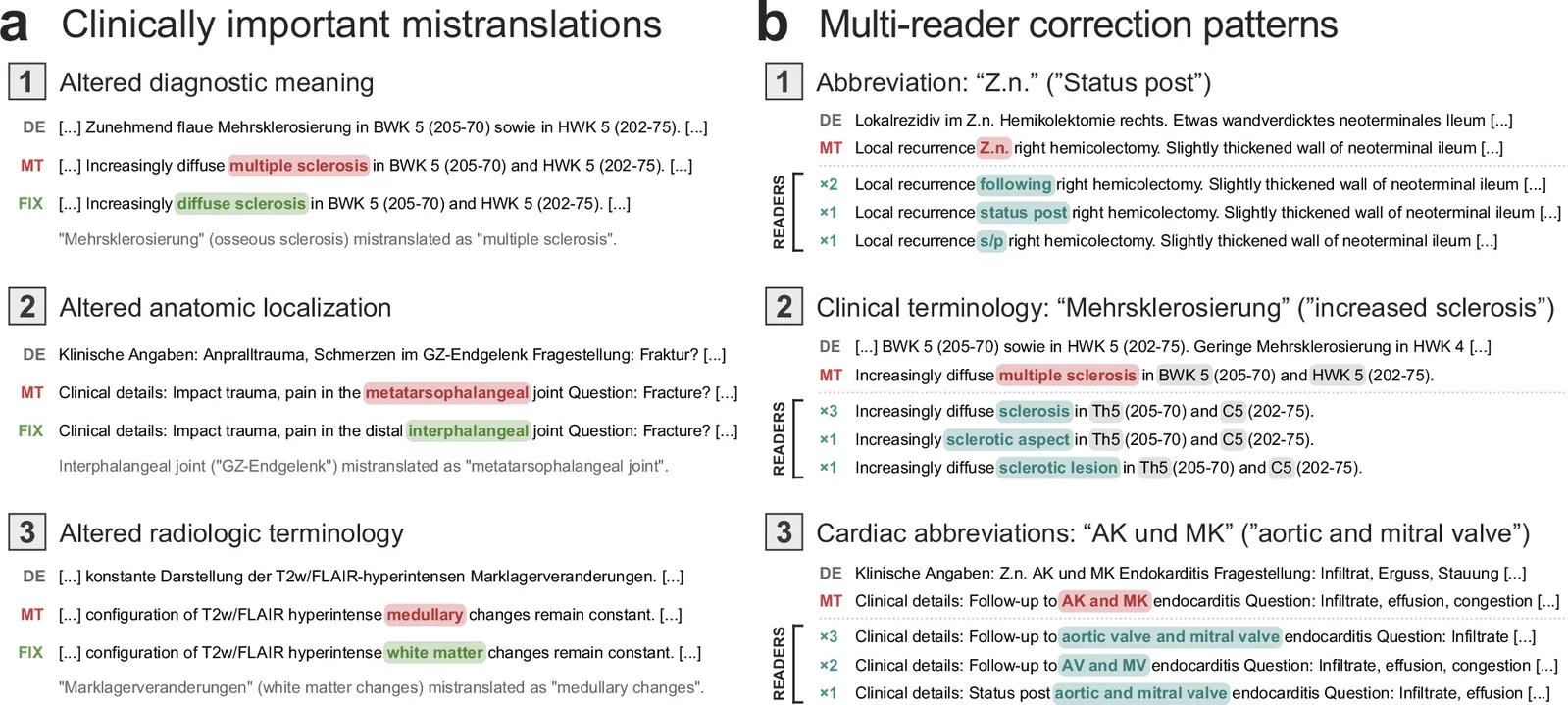
Representative clinically important mistranslations and multi-reader correction patterns. **a** Three representative clinically important mistranslations in the DeepL output, with excerpts from the German source text (DE), MT, and expert correction (FIX). Examples illustrate altered diagnostic meaning, anatomy, and radiologic terminology. **b** Examples of recurrent terms or abbreviations for which multiple radiologists edited the same machine-translated phrase; the number before each correction indicates how many readers made that correction. Gray boxes denote abbreviation-related translation errors that were also revised by the radiologists. Text excerpts are truncated for display

Most DeepL translations were rated good or excellent (339 of 520, 65.2%), and the mean quality score was 3.73 of 5.0 (Fig. 3 and Table 1). Expert review also identified clinically important mistranslations involving diagnosis, anatomy, and radiologic terminology (Fig. 4). Representative examples included translation of “Mehrsklerosierung” as “multiple sclerosis”, mistranslation of an interphalangeal joint as a metatarsophalangeal joint, and mistranslation of white matter changes as medullary changes. In shared reports, readers often corrected the same source error, although the wording of individual corrections varied. Selected recurrent mistranslation patterns and multi-reader correction frequencies are summarized in Supplementary Table S4.

### Reader agreement and contextual benchmark

**Fig. 5:**
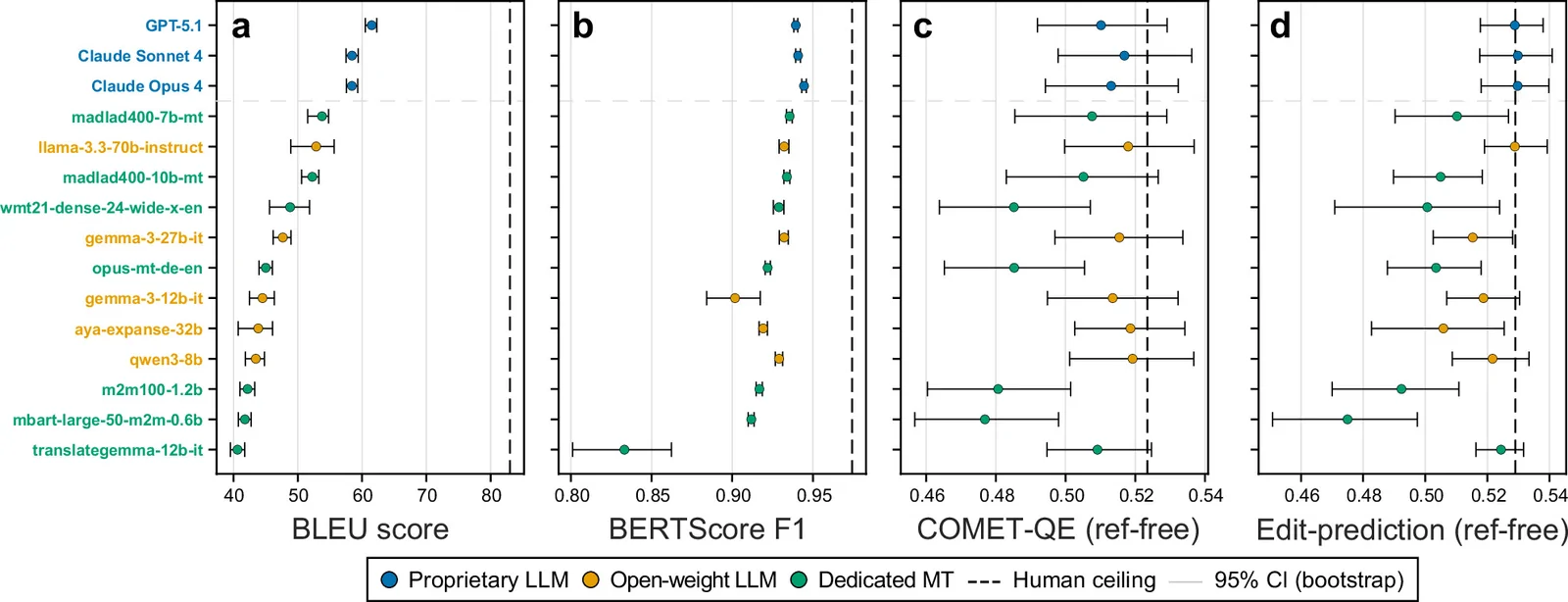
Contextual benchmark of selected translation-system configurations. Systems are grouped as proprietary LLMs, open-weight LLMs, dedicated MT systems, and the inter-reader human ceiling. Panels show (**a**) BLEU, (**b**) BERTScore F1, (**c**) COMET-QE, and (**d**) reference-free edit-prediction score. Error bars indicate 95% bootstrap confidence intervals where applicable. The dashed vertical line denotes the inter-reader human ceiling, defined as agreement among expert-corrected reader references. BLEU, Bilingual evaluation understudy; QE, Quality estimation

Agreement for ordinal quality ratings across shared reports was low (Krippendorff α = 0.078, *p* = 0.006; intraclass correlation coefficient = 0.096), indicating substantial variability in how readers assigned severity scores to the same translations (Table 2). Agreement for expert corrections was high (pairwise BLEU = 83.0, *p* < 0.001), indicating closer agreement in the text that readers changed. In the contextual benchmark, system rankings were stable across expert-corrected references. In variance decomposition of BLEU scores, system quality explained 99.5% of the variance, reader reference explained 0.3%, and the system-by-reader interaction explained 0.2%; Kendall coefficient of concordance was 0.997 (Table 2). In descriptive reference-based benchmark scoring, proprietary LLMs had the highest point estimates (Fig. 5). GPT-5.1 had the highest BLEU score (61.5), followed by Claude Sonnet 4 and Claude Opus 4, but all systems remained below the inter-reader human ceiling of 85.8. Exploratory categorized error analyses of representative contextual benchmark systems are provided in Supplementary Figs. S1 and S2. Across the representative contextual benchmark systems evaluated on the 30 shared reports, recurrent error categories included abbreviation/acronym errors, terminology errors, anatomy/structure errors, and diagnosis/finding errors; laterality, negation/uncertainty, and follow-up/recommendation errors were less frequent but still observed.

**Table 2:** Reader agreement and contextual benchmark stability.

Parameter	Estimate
Krippendorff α for quality scores	0.078 (*p* = 0.006)
Intraclass correlation coefficient for quality scores	0.096
Pairwise BLEU between reader corrections	83.0 (*p* < 0.001)
BLEU variance explained by system	99.5%
BLEU variance explained by reader reference	0.3%
BLEU variance explained by system x reader interaction	0.2%
Kendall coefficient of concordance, W	0.997
Highest contextual benchmark BLEU score	GPT-5.1, 61.5
Inter-reader human ceiling BLEU score	85.8

Agreement metrics were calculated on the shared multi-reader reports. Pairwise “bilingual evaluation understudy” (BLEU) represents the mean of per-report pairwise BLEU scores, whereas the inter-reader human ceiling was calculated using corpus-level BLEU aggregation. Variance decomposition and Kendall coefficient of concordance summarize benchmark stability across available expert-corrected references

### Report-level identification of translations requiring review

In the conservative primary review-trigger screen, 18 of 30 shared reports were review-positive because at least one reader assigned a quality score of 2 or lower. Under this safety-oriented definition, no evaluated automated method significantly discriminated reports requiring review (AUC range, 0.514–0.653; *p* range, 0.100–0.920; Supplementary Table S5). CIs were wide, reflecting the modest shared-report denominator. In the stricter sensitivity analysis requiring at least two readers to assign a quality score of 2 or lower, eight of 30 reports were classified as review-positive. Conventional metrics and mean edit rate again showed limited discrimination (AUC range, 0.500–0.636; *p* range, 0.280–1.000; Supplementary Table S5). The two reference-free LLM judges showed moderate discrimination in this stricter analysis (Claude Sonnet 4: AUC, 0.790; 95% CI: 0.597–0.956; *p* = 0.005; GPT-5.1: AUC, 0.773; 95% CI: 0.588–0.933; *p* = 0.015), whereas conventional metrics remained limited (Supplementary Table S5).

### Segment-level edit prediction

**Fig. 6:**
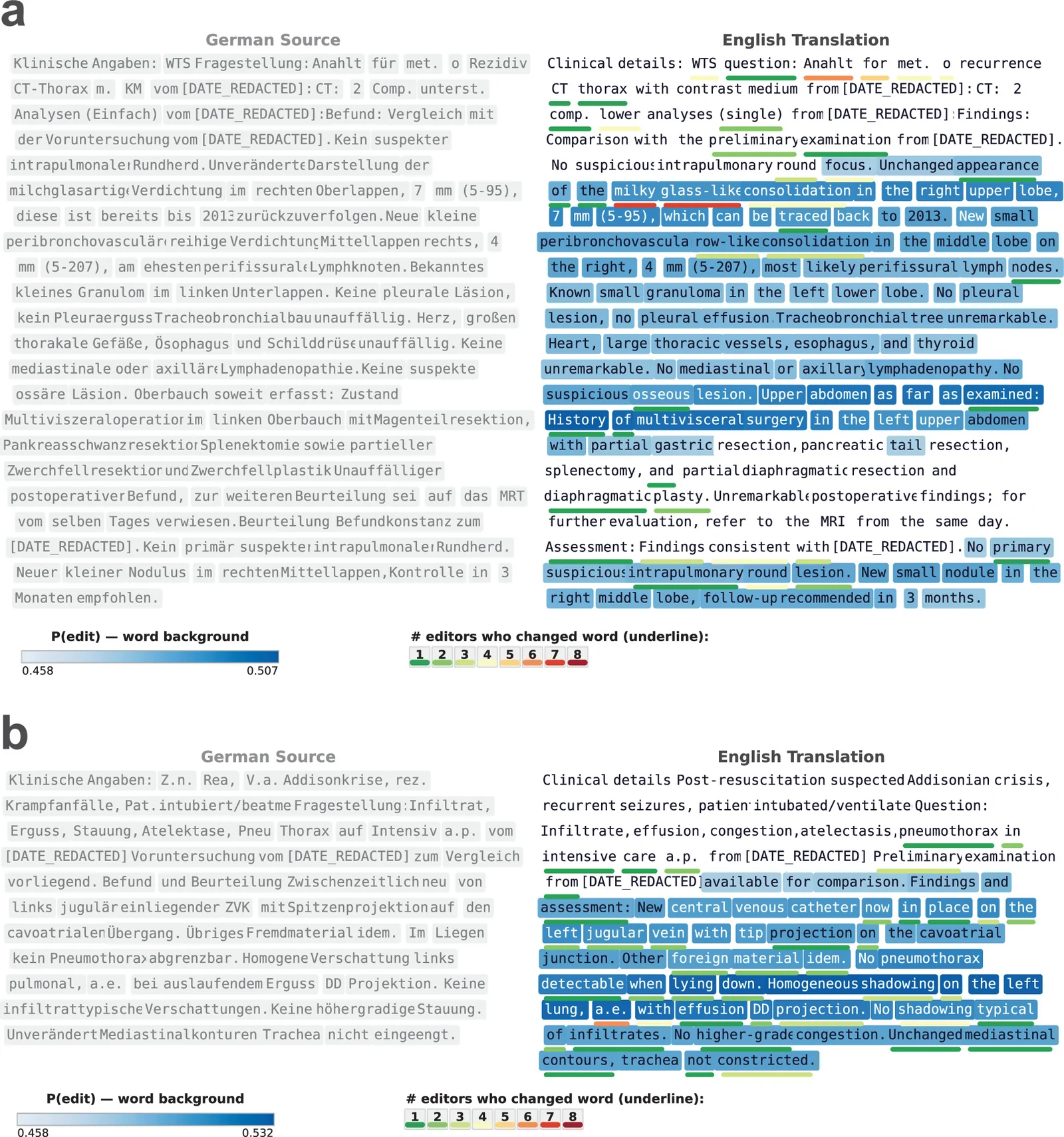
Representative segment-level localization examples from the correction-based edit-prediction model. **a**, **b** Show representative held-out shared reports with German source text and DeepL English translation. Word-level background shading indicates model-predicted edit probability, and underline colors indicate how many of the eight radiologists edited the corresponding word or segment

In the held-out shared reports, the baseline 16-token edit-prediction model achieved a concordance index of 0.639 (95% CI: 0.608, 0.669). Performance increased after supervised contrastive pretraining (concordance index, 0.666; 95% CI: 0.636, 0.695), ill-fit augmentation (concordance index, 0.682; 95% CI: 0.649, 0.711), and cross-scale averaging in the final model (concordance index, 0.697; 95% CI: 0.668, 0.724; *p* < 0.001) (Table 3). The final model achieved an AUC of 0.749 (95% CI: 0.699, 0.796) for segments edited by at least four of eight readers and an AUC of 0.731 (95% CI: 0.688, 0.769) for segments edited by at least six of eight readers. Predicted edit probability increased with multi-reader edit consensus; the segment-level Spearman correlation between predicted edit probability and the number of correcting readers was 0.498 (95% CI: 0.427, 0.565; *p* < 0.001) (Supplementary Fig. S3). At the report level, BERTScore showed the largest correlation magnitude with both mean quality score (rho = 0.588, *p* = 0.001) and the number of correcting readers (rho = -0.700, *p* < 0.001) but required reference translations (Table 4). Among reference-free approaches, Claude Sonnet 4 judge scores correlated with the number of correcting readers (rho = -0.489, *p* = 0.001), as did GPT-5.1 judge scores (rho = -0.421, *p* = 0.042). The correction-based edit-prediction model also correlated with the number of correcting readers (rho = 0.489, *p* = 0.009) and, unlike report-level LLM judges, provided segment-level localization. Representative held-out examples showed localization of report segments requiring correction, including segments with high multi-reader edit consensus (Fig. 6).

**Table 3:** Segment-level edit prediction model development

Parameter	Concordance index (95% CI)	AUC for at least 4 of 8 readers (95% CI)	AUC for at least 6 of 8 readers (95% CI)
BCE baseline (16-token windows)	0.639 (0.608, 0.669)	0.705 (0.653, 0.756)	0.679 (0.636, 0.721)
+ Supervised contrastive pretraining	0.666 (0.636, 0.695; *p* = 0.012)	0.737 (0.685, 0.785; *p* = 0.068)	0.713 (0.672, 0.754; *p* = 0.031)
+ Ill-fit augmentation	0.682 (0.649, 0.711; *p* = 0.002)	0.715 (0.660, 0.766; *p* = 0.644)	0.724 (0.682, 0.765; *p* = 0.016)
+ Cross-scale averaging (final model)	0.697 (0.668, 0.724; *p* < 0.001)	0.749 (0.699, 0.796; *p* = 0.064)	0.731 (0.688, 0.769; *p* = 0.017)

All estimates are based on the 30 held-out shared reports, which contained 601 analyzable 16-token segments. Cross-scale averaging combined predictions from 16-token and 32-token windows. The *p*-values are from a two-sided paired bootstrap comparing each model with the binary cross-entropy (BCE) baseline

*AUC* Area under the receiver operating characteristic curve, *CI* Confidence interval

**Table 4:** Report-level comparison of automated and correction-based methods

Parameter	Spearman rho with mean quality score	Spearman rho with number of correcting readers	Requires reference translation	Provides segment localization
BERTScore	0.588 (0.286, 0.787; *p* = 0.001)	-0.700 (-0.862, -0.427; *p* < 0.001)	Yes	No
BLEU	0.428 (0.077, 0.705; *p* = 0.022)	-0.431 (-0.702, -0.097; *p* = 0.019)	Yes	No
chrF++	0.350 (-0.028, 0.665; *p* = 0.068)	-0.506 (-0.759, -0.176; *p* = 0.005)	Yes	No
COMET-QE	0.351 (-0.107, 0.689; *p* = 0.111)	-0.212 (-0.529, 0.153; *p* = 0.256)	No	No
Claude Sonnet 4 judge	0.517 (0.111, 0.776; *p* = 0.017)	-0.489 (-0.728, -0.182; *p* = 0.001)	No	No
GPT-5.1 judge	0.407 (-0.077, 0.757; *p* = 0.104)	-0.421 (-0.738, -0.017; *p* = 0.042)	No	No
Correction-based edit prediction model	-0.367 (-0.686, 0.038; *p* = 0.060)	0.489 (0.037, 0.777; *p* = 0.009)	No	Yes

Correlations were calculated on the 30 shared reports. Signed Spearman rho values are reported to indicate the direction of association. Bootstrap 95% confidence intervals and two-sided *p*-values were derived from the same 2,000-resample bootstrap distribution. The report-level output of the edit-prediction model was defined as the proportion of windows with predicted edit probability above 0.458. The *p*-values test the null hypothesis of no association (Spearman rho = 0)

*BLEU* Bilingual evaluation understudy, *QE* Quality estimation

## Discussion

In this retrospective multi-reader study of German-to-English machine-translated radiology reports, most DeepL translations were rated favorably, but expert review identified clinically important mistranslations involving diagnosis, anatomy, and radiologic terminology. Ordinal quality scores showed low inter-reader agreement, whereas expert corrections were substantially more reproducible. Automated methods did not identify review-positive reports in the conservative primary screen, although LLM judges showed moderate discrimination in a stricter sensitivity analysis. A correction-based edit-prediction model localized text requiring review, with a concordance index of 0.697 and an AUC of 0.731 for segments edited by at least six of eight readers.

These findings are clinically relevant because radiology reports are dense clinical documents in which small wording changes may alter the meaning of a diagnosis, anatomic location, or follow-up recommendation. In cross-border care, teleradiology, and international referral pathways, MT may therefore be useful for access and efficiency, but average translation quality alone is not sufficient to establish clinical safety. Prior work has suggested that MT can support medical communication and that contemporary LLMs have improved general translation performance [3–5]. Our findings extend this evidence to radiology by showing that errors requiring expert review can persist even when most translations are rated good or excellent.

The low agreement for ordinal quality scores highlights a practical challenge for translation oversight. A single global rating is easy to collect, but it may reflect reader calibration, tolerance for stylistic variation, and perceived clinical severity rather than a stable reference standard. By contrast, reader corrections were more reproducible, suggesting that experts more consistently agreed on the wording that required change than on the numerical severity assigned to the entire translation. For clinical quality assurance, this distinction is important because a correction directly identifies the part of the report that may require review. It also supports a human-in-the-loop framing in which automated systems prioritize text for expert verification rather than replace clinical oversight.

DeepL was selected a priori as the clinically evaluated translation workflow because it represented a predefined and practically available German-to-English MT pipeline. To contextualize this workflow, we additionally benchmarked 51 contemporary MT and LLM-based translation-system configurations against expert-corrected DeepL outputs. Proprietary LLMs had the highest reference-based point estimates and remained below the inter-reader human ceiling, consistent with recent work showing strong multilingual translation performance of LLMs [4, 5]. However, the benchmark references were expert post-edits of DeepL output rather than independently generated de novo reference translations. Systems that produce wording closer to DeepL-style phrasing may therefore be structurally advantaged. Accordingly, the benchmark should be interpreted as a contextual comparison of similarity to clinically corrected DeepL-style translations, whereas the primary clinical safety findings refer to the predefined DeepL workflow evaluated by radiologists.

The report-level screening results further separate aggregate benchmark quality from clinical triage. The conservative primary endpoint was intentionally safety-oriented: one poor or unusable expert rating was sufficient to classify a report as requiring review. This definition may overidentify translations requiring review, particularly given the low inter-reader agreement for ordinal quality scores, but it reflects a cautious workflow in which a single potentially consequential concern should not be ignored. Conventional metrics and reference-free quality estimation did not discriminate review-positive reports in the conservative primary screen, in which one reader flag was sufficient to classify a report as review-positive. This finding is consistent with the known limitation that metrics optimized for average text similarity may miss rare but clinically meaningful errors [17, 18]. In the stricter sensitivity analysis, Claude Sonnet 4 and GPT-5.1 showed moderate discrimination, suggesting that LLM judges may capture some clinically relevant signals when the definition of clinical importance requires greater reader consensus. However, these approaches still operate primarily at the report level and do not by themselves indicate which wording should be checked.

Correction-based segment-level localization may therefore be a promising investigational quality-assurance output. In this study, the model assigned higher edit probabilities to segments corrected by more readers and achieved moderate discrimination for segments edited by at least four or at least six readers. This output should be interpreted as a research-stage review-priority signal rather than as a deployable triage tool or autonomous safety filter. Before clinical implementation, prospective workflow studies are needed to determine whether displaying such localization reduces review time, improves reviewer accuracy, and avoids missed clinically important mistranslations. The same concept may also apply to other generated radiology text, including AI-generated summaries, patient-facing explanations, structured report transformations, and LLM-generated draft reports [8, 21–23]. Recent blinded evidence in lumbar spine MRI reporting showed that radiologist-written reports still outperformed LLM-generated reports in quality and structure, although some AI-generated reports were difficult to distinguish from human-written reports, further supporting the need for expert oversight of fluent AI-generated radiology text [23].

This study has limitations. First, we evaluated one high-resource language pair, German to English, using reports from a single institution. Performance and error types may differ for lower-resource languages, different reporting conventions, or other healthcare systems. Second, DeepL served as the predefined clinically evaluated translation workflow, whereas the broader benchmark was designed to contextualize this workflow against other MT and LLM-based systems. Because the benchmark used expert post-edits of DeepL output as reference translations, it primarily reflects similarity to clinically corrected DeepL-style translations and may favor systems that are lexically or stylistically close to DeepL. Future studies should directly compare multiple translation systems using independent radiologist review of each system output. Third, the multi-reader shared set was modest in size, with 30 reports and 601 analyzable segments. This limited power for binary report-level screening analyses and increased uncertainty around the sensitivity analysis. Fourth, both the report-level review-trigger endpoint and the segment-level edit-prediction model were based on expert ratings or corrections rather than adjudicated downstream patient outcomes. They therefore identify translations and text segments that may require expert review, not direct patient harm, diagnostic error, or clinical impact. Finally, the study was retrospective and did not test whether segment-level localization reduces review time, improves reviewer accuracy, or changes clinical workflow in prospective use. The edit-prediction model should therefore be considered investigational and requires prospective workflow validation before clinical implementation.

In conclusion, German-to-English machine-translated radiology reports were often rated favorably, but translations containing errors requiring expert review persisted. Expert corrections were more reproducible than ordinal quality scores and supported segment-level localization of text requiring human review. These findings support human-in-the-loop translation oversight rather than fully automated clinical use, and prospective workflow studies are needed to determine whether correction-based localization improves the safety and efficiency of radiology report translation.

## Supplementary information

**Additional file: Table S1** Overview of machine-translation and LLM systems used in the contextual benchmark. **Table S2** Prompting and inference settings for LLM-based translation, LLM-as-judge screening, and error-annotation analyses. **Table S3** Inter-reader edit agreement by window size. **Table S4** Selected recurrent mistranslation patterns in shared reports. **Table S5** Report-level discrimination of translations requiring review. **Fig. S1** Categorized error profiles of representative translation systems in the contextual benchmark. Stacked bars show categorized error counts across the 30 shared reports for five representative systems. Bars are stratified by error category; numbers indicate total error counts, and whiskers denote bootstrap 95% confidence intervals. Error categories included mistranslation, omission, addition, untranslated text, abbreviation, number/unit/date, anatomic localization, altered diagnostic meaning, terminology, grammar/fluency, and formatting. This exploratory analysis contextualizes error profiles of selected benchmark systems and should not be interpreted as a direct clinical safety ranking of all translation systems. **Fig. S2** Error type and frequency by clinical axis. (**a**) Overall error frequencies by category, shown as per-report error rates for representative commercial models and open-weight models. Category labels indicate pooled error counts across the included systems. (**b**) Heatmap of clinically oriented error categories by individual system, including terminology, anatomy/structure, diagnosis/finding, follow-up/recommendation, negation/uncertainty, and laterality; numbers indicate categorized error counts, and the rightmost column shows row totals. (**c**) Overall error burden per report for each representative system. This supplementary exploratory analysis was added to illustrate recurrent error patterns and relative error burden across representative systems. **Fig. S3** Predicted edit probability and multi-reader consensus severity. (**a**) Distribution of segment-level predicted edit probability according to the number of correcting readers. (**b**) Mean predicted edit probability according to the number of correcting readers. Predictions are based on cross-scale averaging of the 16-token and 32-token models.

## Data Availability

The clinical text data used in this study are not publicly available because they originate from institutional radiology reports and remain subject to data protection and institutional governance restrictions, even after de-identification. Aggregated data supporting the main findings are included in the article and its supplementary material. Additional de-identified derived data may be available from the corresponding author on reasonable request and subject to institutional approval.

## Abbreviations

API: Application programming interface
BLEU: Bilingual evaluation understudy
COMET: Crosslingual optimized metric for evaluation of translation
COMET-QE: COMET quality estimation
CT: Computed tomography
LLM: Large language model
MT: Machine translation
