# Supplementary material for: Fluent but not fail-safe: machine-translated radiology reports still need expert review
Source: Eur Radiol Exp. 2026 Oct 14;10:158. doi: 10.1186/s41747-026-00827-7 (PMC13669263; doi:10.1186/s41747-026-00827-7)
Supplement: Supplementary file 1 [file 41747_2026_827_MOESM1_ESM.docx]

**Supplementary material**

**Table S1** Overview of machine-translation and LLM systems used in the contextual benchmark

| **Model** | **Parameters** | **Access** | **Precision** |
| --- | --- | --- | --- |
| **Proprietary API-based LLMs^†^** |  |  |  |
| Claude Opus 4 (claude-opus-4-20250514) | — | Anthropic API | API-hosted |
| Claude Sonnet 4 (claude-sonnet-4-20250514) | — | Anthropic API | API-hosted |
| GPT-5.1 | — | OpenAI API | API-hosted |
| **Open-weight LLMs** |  |  |  |
| CohereForAI/aya-expanse-32b | 32B | HuggingFace | 4-bit NF4^‡^ |
| CohereForAI/aya-expanse-8b | 8B | HuggingFace | 4-bit NF4^‡^ |
| Qwen/Qwen2.5-32B-Instruct | 32B | HuggingFace | 4-bit NF4^‡^ |
| Qwen/Qwen2.5-7B-Instruct | 7B | HuggingFace | 4-bit NF4^‡^ |
| Qwen/Qwen3-32B | 32B | HuggingFace | 4-bit NF4^‡^ |
| Qwen/Qwen3-8B | 8B | HuggingFace | 4-bit NF4^‡^ |
| google/gemma-3-12b-it | 12B | HuggingFace | 4-bit NF4^‡^ |
| google/gemma-3-27b-it | 27B | HuggingFace | 4-bit NF4^‡^ |
| meta-llama/Llama-3.1-8B-Instruct | 8B | HuggingFace | 4-bit NF4^‡^ |
| meta-llama/Llama-3.3-70B-Instruct | 70B | HuggingFace | 4-bit NF4^‡^ |
| microsoft/Phi-4 | 14B | HuggingFace | 4-bit NF4^‡^ |
| mistralai/Mistral-Small-Instruct-2409 | 22B | HuggingFace | 4-bit NF4^‡^ |
| mistralai/Mixtral-8x7B-Instruct-v0.1 | 46.7B (MoE) | HuggingFace | 4-bit NF4^‡^ |
| utter-project/EuroLLM-9B-Instruct | 9B | HuggingFace | 4-bit NF4^‡^ |
| **Dedicated machine-translation models^§^** |  |  |  |
| Helsinki-NLP/opus-mt-de-en | ≈74M | HuggingFace | FP16 |
| Unbabel/Tower-Plus-9B | 9B | HuggingFace | 4-bit NF4^‡^ |
| alirezamsh/small100 | 330M | HuggingFace | FP16 |
| facebook/m2m100_1.2B | 1.2B | HuggingFace | FP16 |
| facebook/mbart-large-50-many-to-many-mmt | 611M | HuggingFace | FP16 |
| facebook/nllb-200-3.3B | 3.3B | HuggingFace | FP16 |
| facebook/nllb-200-distilled-1.3B | 1.3B | HuggingFace | FP16 |
| facebook/nllb-200-distilled-600M | 600M | HuggingFace | FP16 |
| facebook/wmt19-de-en | ≈580M | HuggingFace | FP16 |
| facebook/wmt21-dense-24-wide-x-en | 4.7B | HuggingFace | FP16 |
| google/madlad400-10b-mt | 10B | HuggingFace | FP16 |
| google/madlad400-7b-mt | 7.2B | HuggingFace | FP16 |
| google/translategemma-12b-it | 12B | HuggingFace | 4-bit NF4^‡^ |
| google/translategemma-27b-it | 27B | HuggingFace | 4-bit NF4^‡^ |
| tencent/HY-MT1.5-1.8B | 1.8B | HuggingFace | 4-bit NF4^‡^ |
| tencent/HY-MT1.5-7B | 7B | HuggingFace | 4-bit NF4^‡^ |
| tencent/Hunyuan-MT-7B | 7B | HuggingFace | 4-bit NF4^‡^ |
| google/madlad400-3b-mt* | 3B | HuggingFace | FP16 |
| haoranxu/ALMA-13B-R* | 13B | HuggingFace | 4-bit NF4^‡^ |
| haoranxu/ALMA-7B-R* | 7B | HuggingFace | 4-bit NF4^‡^ |

Machine-translation and large language model (LLM) systems used in the contextual benchmark. The table lists model backbones, access modalities, parameter counts, and inference precision for the systems evaluated in the contextual benchmark. The 51 reported translation-system configurations comprised nine proprietary API-based LLM configurations, 14 open-weight LLM configurations, and 28 dedicated machine-translation configurations. HuggingFace model identifiers are reported as repository names to support reproducibility. All HuggingFace models were run deterministically using greedy decoding. API-hosted models were accessed on 2026-03-21 through their respective provider APIs; for these models, inference precision was not user-controlled. ^†^Three prompt variants were evaluated for each proprietary API-based LLM, resulting in nine proprietary LLM configurations. ^§^Dedicated machine-translation configurations comprised 22 sentence-level and paragraph-level configurations from seq2seq encoder-decoder MT models and six paragraph-level configurations from decoder-only MT models. Configurations producing degenerate outputs were not retained for the reported benchmark comparison. *Models marked with an asterisk were included in exploratory or supplementary analyses and were not part of the 51 reported translation-system configurations. ^‡^*NF4* NormalFloat 4-bit quantization, *MT* Machine translation, *MoE* Mixture of experts.

## Table S2 Prompting and inference settings for LLM-based translation, LLM-as-judge screening, and error-annotation analyses

| **Analysis component** | **Systems** | **Prompting strategy** | **Prompt variants** | **Inference settings** | **Output format** |
| --- | --- | --- | --- | --- | --- |
| Proprietary LLM translation benchmark | GPT-5.1; Claude Sonnet 4; Claude Opus 4 | German source reports were translated using radiology-specific or general German-to-English translation instructions. | Three variants: P1 domain-expert, P2 minimal, P3 detailed-clinical | API-based inference; temperature = 0; deterministic decoding where supported; inference date: 2026-03-21 | English translation only |
| Open-weight LLM translation benchmark | 14 open-weight LLM configurations listed in Table S1 | German source reports were translated using the domain-expert radiology prompt. | One variant: P1 domain-expert | Local HuggingFace inference; temperature = 0; deterministic greedy decoding; inference date: 2026-03-21 | English translation only |
| Dedicated machine-translation benchmark | 28 dedicated MT configurations listed in Table S1 | Dedicated MT models were run using model-specific German-to-English translation interfaces or source-target templates. | Not applicable | Local HuggingFace inference; deterministic greedy decoding; inference date: 2026-03-21 | English translation only |
| Reference-free LLM-as-judge screening | Claude Sonnet 4 (claude-sonnet-4-20250514); GPT-5.1 | Each model received the German source report and the corresponding DeepL English translation and was instructed to compare both texts, assign a 5-point quality score, provide a brief explanation, and return a corrected English translation if needed. No expert reference translation was provided. | One fixed radiology quality-assessment prompt, provided in Appendix A as P4 | API-based inference; temperature = 0; deterministic decoding where supported; no user-specified seed; default provider settings otherwise; inference date: 2026-03-21 | XML with <quality>, <quality_label>, <comment>, and <corrected_translation> fields. The numeric <quality> value was parsed as the report-level LLM judge score. For review-trigger discrimination analyses, scores were directionally aligned so that higher values indicated greater likelihood of requiring review. |
| Categorized error analysis | Representative benchmark systems evaluated on the 30 shared reports | MQM-style radiology German-to-English translation-adequacy guideline comparing German source text, model output, and eight radiologist reference translations. | One objective annotation guideline | Temperature = 0; deterministic decoding where supported | JSONL, one object per error span |

Prompting and inference settings for large language model (LLM)-based translation, reference-free LLM-as-judge screening, dedicated machine-translation models, and exploratory error annotation. The table summarizes the model-group-specific prompting strategy, prompt variants, inference date, temperature setting, deterministic decoding strategy, and output format. Proprietary API-based LLMs were evaluated with three prompt variants, open-weight LLMs with the domain-expert radiology prompt, and dedicated machine-translation models with model-specific German-to-English translation interfaces or templates. Reference-free LLM-as-judge screening was performed without access to expert-corrected reference translations; each judge received only the German source report and the corresponding DeepL English translation. All LLM-based inference was performed with temperature = 0 and deterministic decoding where supported. Complete LLM prompt texts, including the LLM-as-judge prompt, are provided in Appendix A. The MQM-style radiology German-to-English error-annotation guideline used for the exploratory categorized error analysis is provided in Appendix B. *MQM* Multidimensional Quality Metrics, *MT* Machine translation.

**Table S3** Inter-reader edit agreement by window size

| **Parameter** | **Number of segments** | **Base editing rate** | **Z statistic** | ***p-*value** |
| --- | --- | --- | --- | --- |
| 8-token windows | 1143 | 59.5% | 5.1 | < 0.001 |
| 16-token windows | 601 | 66.7% | 4.4 | < 0.001 |
| 32-token windows | 303 | 72.8% | 2.9 | 0.003 |
| 64-token windows | 159 | 77.1% | 0.6 | 0.300 |
| 128-token windows | 84 | 82.6% | -0.7 | 0.780 |

Block-permutation testing preserved each reader’s marginal edit rate. The 16-token window size was used as the primary resolution because it retained significant multi-reader signal while providing finer localization than larger windows.

**Table S4** Selected recurrent mistranslation patterns in shared reports

| **Parameter** | **DeepL output** | **Representative expert correction** | **Type** | **Total occurrences, *n*** | **Mean readers correcting, *n*** |
| --- | --- | --- | --- | --- | --- |
| 2 Ebenen | 2E | 2 planes | Abbreviation | 13 | 3.2 |
| Zustand nach | Z.n. | Status post | Abbreviation | 6 | 2.0 |
| Mehrsklerosierung | multiple | sclerosis / deletion of “multiple” | Clinical terminology | 15 | 4.0 |
| Marklager | medullary | white matter | Clinical terminology | 8 | 4.0 |
| dargestellt | visualized | captured | Radiology terminology | 15 | 8.0 |
| Beurteilung | assessment | impression | Stylistic preference | 19 | 1.2 |

Patterns were extracted from the shared reports because multi-reader correction frequencies could be quantified. High mean reader counts indicate consensus edits; low counts indicate more variable stylistic preferences.

**Table S5** Report-level discrimination of translations requiring review

| **Method** | **AUC (95% CI)** | ***p-*value** |
| --- | --- | --- |
| **Primary review-trigger endpoint (≥ 1 reader score ≤ 2)** | | |
| BLEU | 0.560 (0.330, 0.774) | 0.606 |
| chrF++ | 0.514 (0.279, 0.735) | 0.920 |
| BERTScore | 0.602 (0.375, 0.805) | 0.366 |
| COMET-QE | 0.653 (0.435, 0.846) | 0.163 |
| mean edit rate | 0.546 (0.312, 0.768) | 0.688 |
| Claude Sonnet 4 | 0.653 (0.493, 0.799) | 0.100 |
| GPT-5.1 | 0.606 (0.428, 0.775) | 0.247 |
| **Sensitivity endpoint (≥ 2 readers score ≤ 2)** | | |
| BLEU | 0.551 (0.296, 0.792) | 0.690 |
| chrF++ | 0.506 (0.250, 0.761) | 0.981 |
| BERTScore | 0.614 (0.345, 0.858) | 0.374 |
| COMET-QE | 0.636 (0.324, 0.926) | 0.280 |
| mean edit rate | 0.500 (0.245, 0.750) | 1.000 |
| Claude Sonnet 4 | 0.790 (0.597, 0.956) | 0.005 |
| GPT-5.1 | 0.773 (0.588, 0.933) | 0.015 |

Report-level discrimination of translations requiring review. Report-level discrimination was evaluated in the 30 shared reports reviewed by all eight radiologists. The primary review-trigger endpoint defined a report as review-positive if at least one reader assigned a quality score of 2 or lower. The sensitivity endpoint required at least two readers to assign a quality score of 2 or lower. Area under the receiver operating characteristic curve (AUC) values are shown with bootstrap 95% confidence intervals (CI) in parentheses. The *p*-values were obtained by permutation testing with 10,000 permutations. *QE* Quality estimation.

**Fig. S1** Categorized error profiles of representative translation systems in the contextual benchmark. Stacked bars show categorized error counts across the 30 shared reports for five representative systems. Bars are stratified by error category; numbers indicate total error counts, and whiskers denote bootstrap 95% confidence intervals. Error categories included mistranslation, omission, addition, untranslated text, abbreviation, number/unit/date, anatomic localization, altered diagnostic meaning, terminology, grammar/fluency, and formatting. This exploratory analysis contextualizes error profiles of selected benchmark systems and should not be interpreted as a direct clinical safety ranking of all translation systems.

**Fig. S2** Error type and frequency by clinical axis. **a** Overall error frequencies by category, shown as per-report error rates for representative commercial models and open-weight models. Category labels indicate pooled error counts across the included systems. **b** Heatmap of clinically oriented error categories by individual system, including terminology, anatomy/structure, diagnosis/finding, follow-up/recommendation, negation/uncertainty, and laterality; numbers indicate categorized error counts, and the rightmost column shows row totals. **c** Overall error burden per report for each representative system. This supplementary exploratory analysis was added to illustrate recurrent error patterns and relative error burden across representative systems.

**Fig. S3** Predicted edit probability and multi-reader consensus severity. **a** Distribution of segment-level predicted edit probability according to the number of correcting readers. **b** Mean predicted edit probability according to the number of correcting readers. Predictions are based on cross-scale averaging of the 16-token and 32-token models.

**Appendix A**

**Prompt texts for LLM-based translation and reference-free LLM-as-judge screening**

P1: Domain-expert prompt

You are an expert medical translator specializing in radiology. Translate the following German radiology report into English. Preserve all medical terminology, measurements, anatomical references, dates, and report structure exactly. Do not add explanations or commentary. Output only the translated report.

P2: Minimal prompt

Translate the following German text into English. Output only the translation, nothing else.

P3: Detailed-clinical prompt

You are a board-certified radiologist fluent in German and English. Translate the following German radiology report into English. Requirements: (1) Maintain clinical accuracy — do not change the medical meaning. (2) Resolve German medical abbreviations to their standard English equivalents where unambiguous. (3) Preserve the original section structure (e.g. Befund, Beurteilung). (4) Keep all measurements, dates, and anatomical references exact. Output only the translated report.

P4: Reference-free LLM-as-judge prompt

SYSTEM_PROMPT — template:

You are a board-certified radiologist participating in a structured reader study on the quality of machine-translated radiology reports (German → English, translated by DeepL).

## Your task

For each case you will receive:

1. The **original German radiology report**
2. The **automatic English translation** produced by DeepL

You must perform three steps:

### Step 1 — Compare texts

Compare the German original with the automatic English translation. Focus on: accuracy, clinical correctness, preservation of meaning, and detection of potentially dangerous translation errors.

### Step 2 — Rate translation quality

Use the following 5-point Likert scale:

| **Score** | **Label** | **Definition** |
| --- | --- | --- |
| 5 | excellent | Flawless, professionally high-quality medical translation that can be used clinically without any changes |
| 4 | good | High-quality translation with only minimal, non-critical issues |
| 3 | fair | Meaning is mostly correctly conveyed, but several points must be corrected before clinical use |
| 2 | poor | Significant errors that compromise clinical correctness; meaning partially preserved but relevant risk of misinterpretation |
| 1 | useless | The translation does not convey the clinical meaning; high risk of patient-endangering errors |

### Step 3 — Correct the English translation

- Make corrections **only in the English text**.
- Corrections should address:
  - Terminology
  - Grammar and sentence structure
  - Elimination of ambiguities
  - Correction of clinically relevant translation errors
- If the translation is flawless (score 5), return it unchanged.
- Do NOT add explanations, annotations, or markup to the corrected text. Return only the corrected English report text.

## Output format

Respond with EXACTLY this XML structure — nothing else before or after:

<quality>INTEGER 1-5</quality>

<quality_label>LABEL</quality_label>

<comment>Brief explanation of your assessment and any issues found. If no issues, write "No issues identified."</comment>

<corrected_translation>The full corrected English translation. If score is 5, copy the original translation unchanged.</corrected_translation>

USER_PROMPT — template:

Evaluate the following case:

=== GERMAN ORIGINAL ===
{german}

=== DEEPL ENGLISH TRANSLATION ===
{english}

Provide your evaluation in the XML format specified in your instructions.

Prompt assignment

The three prompt variants were evaluated for proprietary API-based LLMs. Open-weight LLMs were evaluated with the domain-expert prompt only. Dedicated machine-translation models were not prompted with free-text clinical instructions; they were run using their model-specific German-to-English translation interface or source-target template.

**Appendix B**

**MQM-style error-annotation guideline for radiology DE-to-EN translation errors**

Categorized error analysis was performed using an objective MQM-style radiology DE-to-EN translation-adequacy guideline. The task was to classify the type and locus of translation errors, not to rate clinical severity or patient risk. For each case and model hypothesis, the annotator compared the German source report, the model output, and eight radiologist reference translations. A model output was counted as erroneous only when it deviated from the source meaning or from acceptable English radiology rendering and was not supported by any of the eight radiologist references. Redaction tokens were not counted as errors.

Each error span was assigned exactly one category according to a fixed priority order: abbreviation, number/unit/date, anatomic localization, altered diagnostic meaning, untranslated text, omission, addition, radiologic terminology, other mistranslation, grammar/fluency, and formatting. When more than one category could apply, the higher-priority category was assigned. The output was structured as JSONL with one object per distinct error span, including case identifier, model name, error category, German source span, offending English hypothesis span, reference or correction span, and a short explanation.
